# Bradycardia, Renal Failure, Atrioventricular Nodal Blockade, Shock, and Hyperkalemia Syndrome as a Clinical Profile Leading to the Diagnosis of Transthyretin Amyloidosis: A Report of Two Cases

**DOI:** 10.7759/cureus.25444

**Published:** 2022-05-29

**Authors:** Koji Takahashi, Tomoki Sakaue, Shigeki Uemura, Takafumi Okura, Shuntaro Ikeda

**Affiliations:** 1 Department of Cardiology, Yawatahama City General Hospital, Ehime, JPN; 2 Department of Community Emergency Medicine, Ehime University Graduate School of Medicine, Ehime, JPN

**Keywords:** carvedilol, amlodipine, verapamil, transthyretin amyloidosis, brash syndrome

## Abstract

We describe two cases in which the onset of bradycardia, renal failure, atrioventricular (AV) nodal blockade, shock, and hyperkalemia (BRASH) syndrome led to the diagnosis of transthyretin cardiac amyloidosis. In Case 1, BRASH syndrome developed shortly after a therapeutic dose of AV nodal blockers was prescribed for new-onset atrial flutter. BRASH syndrome improved with intravenous dopamine infusion and temporary cardiac pacing. In Case 2, BRASH syndrome developed immediately after bronchopneumonia followed by worsening heart failure, despite no change in medications such as AV nodal blockers. Intravenous injection of calcium dramatically improved BRASH syndrome.

## Introduction

Bradycardia, renal failure, atrioventricular (AV) nodal blockade, shock, and hyperkalemia (BRASH) syndrome, first reported by Farkas et al. in 2016 [1], is initiated by synergistic bradycardia due to the combination of hyperkalemia and AV nodal blocking medications. This is a clinically underrecognized syndrome, and deterioration of renal function, worsening of hyperkalemia, and hemodynamic instability can occur if left untreated. The common precipitant is hypovolemia or medications that promote hyperkalemia or renal injury [1].

Cardiac amyloidosis is characterized by amyloid fibril deposition in the heart as part of systemic amyloidosis. Although more than 30 proteins are known to aggregate as amyloid in vivo, only nine amyloidogenic proteins accumulate in the myocardium to cause significant cardiac disease [2]. Nevertheless, many cases of currently diagnosed cardiac amyloidosis result from fibrils composed of amyloid light chains or amyloid transthyretin (ATTR). ATTR amyloidosis is classified into two forms, wild type and variant type, depending on the presence or absence of genetic mutations in transthyretin. Although considered a rare disease, recent data suggest that cardiac amyloidosis is underappreciated as a cause of common cardiac diseases such as heart failure and atrial fibrillation/flutter.

We herein described two cases in which the onset of BRASH syndrome led to the diagnosis of ATTR cardiac amyloidosis. This is the first report of ATTR cardiac amyloidosis complicated by BRASH syndrome.

## Case presentation

Case 1

**Figure 1:**
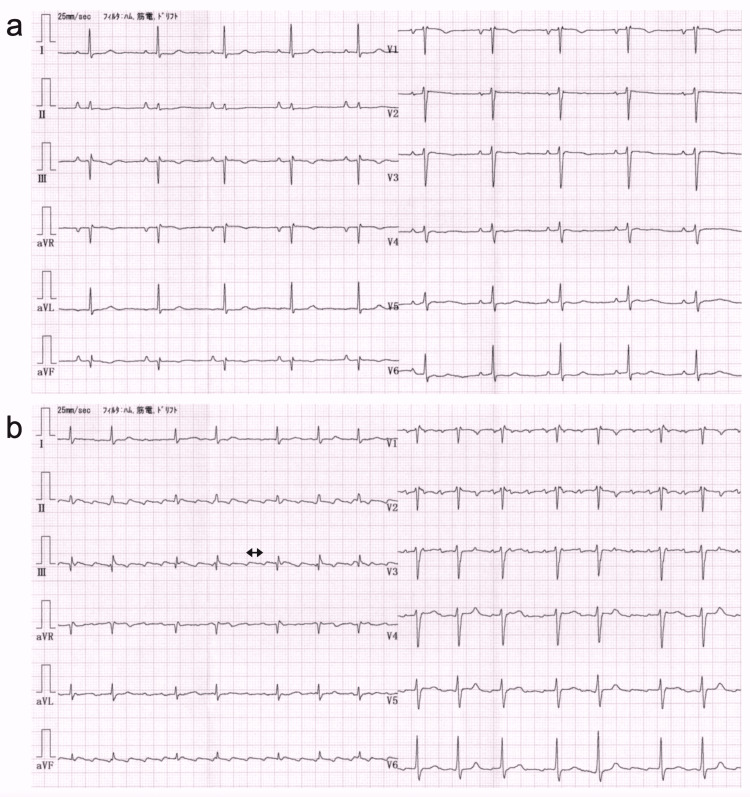
Electrocardiogram (ECG) of Case 1 recorded in another clinic two years (a) and one day (b) before admission, respectively The ECG recorded two years before admission shows sinus rhythm with a heart rate of 60 bpm, abnormal P terminal force, incomplete right bundle branch block, Q waves, negative T waves in leads III and aVF, and poor R-wave progression in the precordial leads. The ECG one day before admission shows atrial flutter with F waves at a rate of 256/min (cycle length, 234 ms) and irregular ventricular rate. The double-headed arrow indicates the cycle length of the F wave.

An 86-year-old Japanese man presented at Yawatahama City General Hospital on foot, with a four-day history of exertional dyspnea and a one-day history of chest discomfort and general malaise. Diseases being treated at another clinic included long-standing hypertension, chronic kidney disease with a serum creatinine level of approximately 1.0 mg/dL and an estimated glomerular filtration rate (eGFR) of 50 mL/min/1.73 m^2^, gastric ulcer after successful eradication of *Helicobacter pylori*, prostatic cancer, and osteoarthritis of the knees. However, none of the patient’s medical history indicates a high index of suspicion for cardiac amyloidoses, such as carpal tunnel syndrome, lumbar spinal stenosis, or biceps tendon rupture. The patient was prescribed oral losartan potassium (50 mg once daily), vonoprazan fumarate (10 mg once daily), bicalutamide (80 mg once daily), mirabegron (50 mg once daily), celecoxib (100 mg twice daily), and LAC-B (1 g three times daily) at the clinic. The patient has been taking these medications for years, with no recent changes in dosage. The electrocardiogram (ECG) recorded in the clinic two years prior revealed normal sinus rhythm (Figure 1a).

One day prior to presentation at Yawatahama City General Hospital, the patient visited the clinic because of exertional dyspnea that persisted for three days. An ECG revealed a new-onset atrial flutter (Figure 1b). Thus, verapamil (40 mg twice daily), carvedilol (1.25 mg once daily), and apixaban (5 mg twice daily) were given. However, the patient complained of chest discomfort and general malaise.

**Figure 2:**
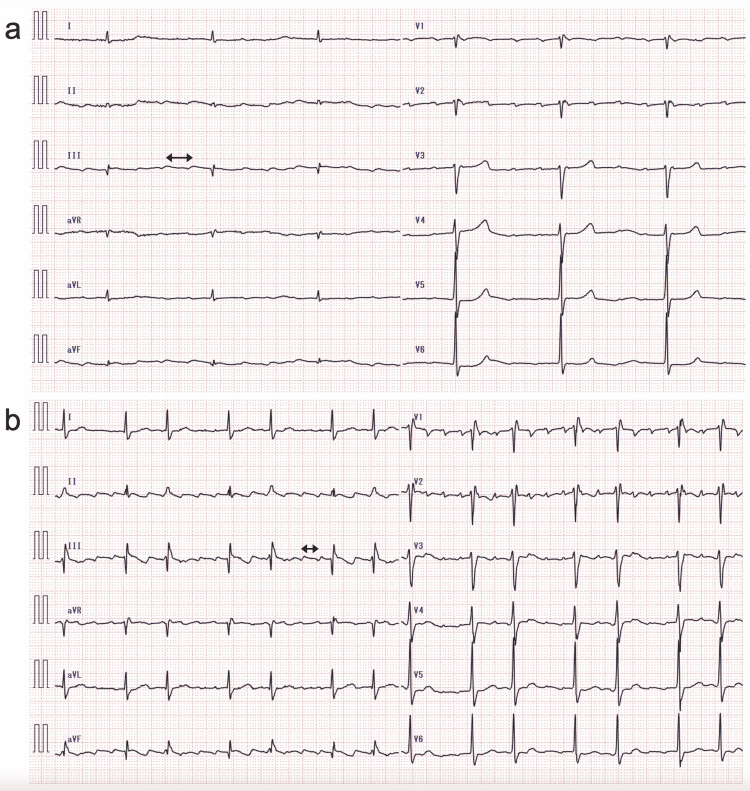
Electrocardiogram (ECG) of Case 1 recorded upon admission (a) and on the third hospital day (b) The ECG recorded upon admission reveals atrial flutter with 4:1 atrioventricular (AV) conduction. Although the cycle length of the F waves was 381 ms and prolonged, the flutter rate was 157/min and the ventricular rate was 39/min. The ECG recorded on the third hospital day shows atrial flutter with F waves at a rate of 239/min (cycle length, 251 ms) and irregular ventricular rate with alternating 2:1 and 4:1 AV conduction. Double-headed arrows indicate the cycle length of the F wave.

**Figure 3:**
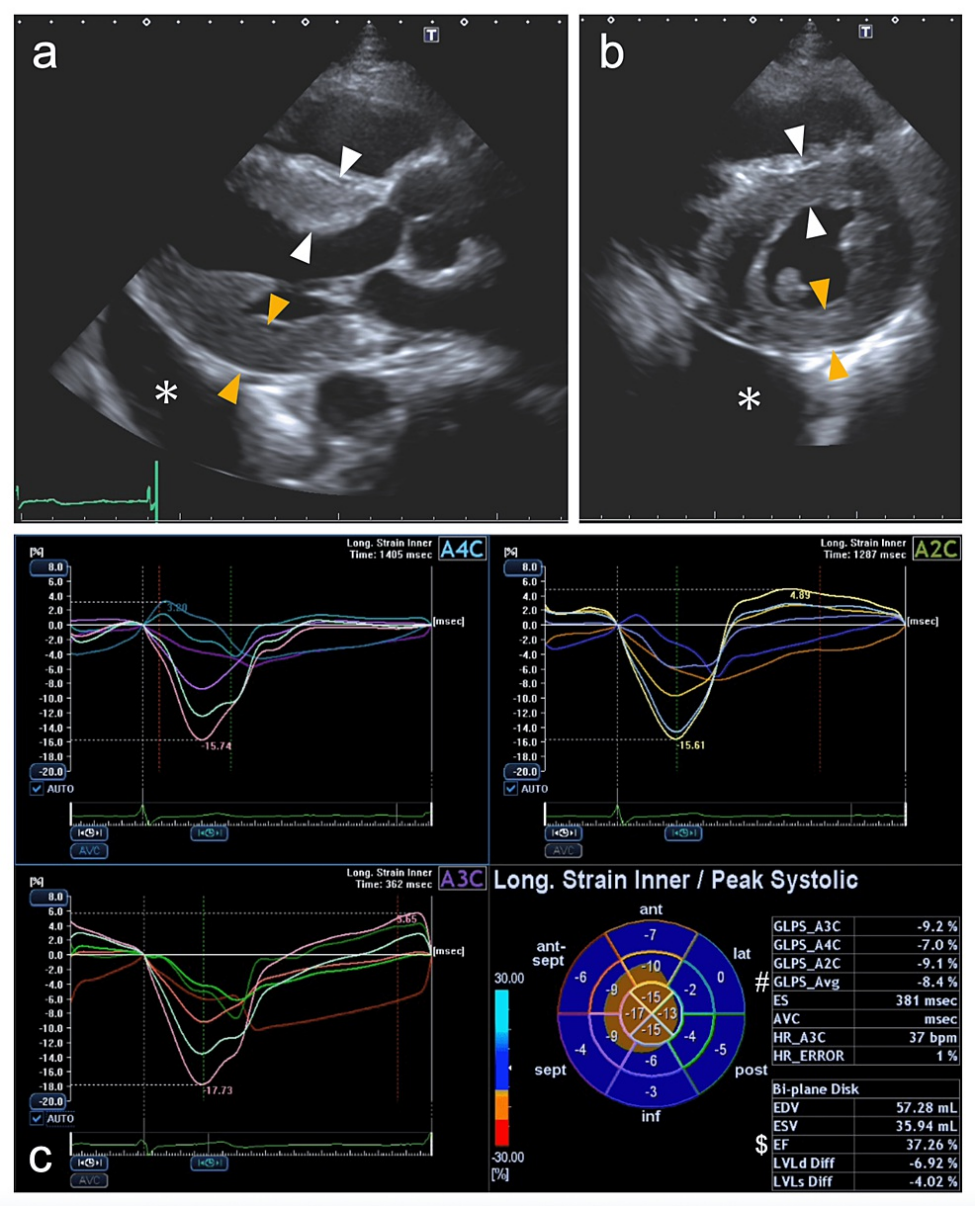
Echocardiograms of Case 1 recorded upon admission A reduced left ventricle with an end-diastolic dimension of 40.8 mm and an increase in the interventricular septum (white arrowheads) and left ventricular (LV) posterior wall (orange arrowheads) of 12.7 and 12.8 mm, respectively, and a normal-sized left atrium with a volume of 38.6 mL/m^2^ are shown (a and b). The bull’s eye map (with the apex at the center of the color-coding map) illustrates segmental longitudinal LV peak systolic strain values of the 16-segment model generated by speckle-tracking analysis of two-dimensional LV images acquired from apical 2-, 3-, and 4-chamber views (A2C, A3C, and A4C, respectively) and shows a reduced LV ejection fraction (EF) of 37.3% (marked with a dollars sign) and LV global longitudinal peak systolic strain (GLS) value, calculated as the mean of these 16 values, of -8.4% (marked with a hash mark) with an LVEF/|GLS| of 4.4, indicating apical sparing (cutoff, >4.1) (c). Asterisks indicate pleural effusion.

Upon arrival at the Yawatahama City General Hospital, the patient had the following vital signs: temperature, 35.9°C; pulse rate, 40 bpm; systemic blood pressure, 117/79 mmHg; oxygen saturation level, 89% on room air. Blood tests revealed an elevated high-sensitivity cardiac troponin I value and brain natriuretic peptide value, renal dysfunction, and liver injury (Table 1). The patient’s serum potassium level was 5.1 mEq/L. Chest radiography revealed pulmonary congestion and retention of bilateral pleural effusion. ECG revealed atrial flutter with a 4:1 AV conduction ratio and a QRS rate of 39/min (Figure 2a). An echocardiogram demonstrated a reduced left ventricular (LV) cavity with an end-diastolic dimension of 40.8 mm and an increase in the interventricular septum and LV posterior wall of 12.7 and 12.8 mm, respectively (Figure 3). Hypokinetic wall motions in the entire LV with an ejection fraction (LVEF) of 37.3% were accompanied by a global longitudinal strain (GLS) of -8.4% and an LVEF strain ratio (LVEF/|GLS|) of 4.4, indicating apical sparing (cutoff value >4.1) [3]. The left atrium, which had a volume of 38.6 mL/m^2^, was not dilated. Thus, the diagnosis included acute decompensated heart failure caused by suspected cardiac amyloidosis. This is associated with atrial flutter with slow AV node conduction and reduced systolic LV function, impaired liver function secondary to congestive heart failure, and worsening renal function. In addition, we suspected verapamil and carvedilol intoxication. The patient was admitted to the hospital for close monitoring. First, verapamil and carvedilol were discontinued, and a single 20 mg dose of furosemide was administered intravenously. The patient also received oxygen via nasal cannula. The serum potassium level (5.1 mEq/L) was slightly deviated from the reference value (3.6-5.0 mEq/L); however, we did not consider therapeutic intervention (Table 1).

**Table 1:** Laboratory test values on admission and after treatment BNP = brain natriuretic peptide; hs-cTnI = high-sensitivity cardiac troponin I; eGFR = estimated glomerular filtration rate; ALT = alanine amino transaminase; AST = aspartate aminotransaminase; ALP = alkaline phosphatase; PCO_2_ = partial pressure of carbon dioxide oxygen; PO_2_ = partial pressure of oxygen; HCO_3_^-^ = bicarbonate ion *Determined through arterial blood gas analysis at the onset of bradycardia, renal failure, atrioventricular nodal blockade, shock, and hyperkalemia syndrome under oxygen inhalation of 3 L/min via nasal cannula. †Examined through arterial blood gas analysis.

Tests	Case 1		Case 2		Reference value
	On admission	After treatment	On admission	After treatment	
BNP (pg/mL)	158.6		924.7		≤18.4
hs-cTnI (pg/mL)	178.0		111.4		≤18.4
Creatinine (mg/dL)	1.47	0.95	1.38	0.78	Male: 0.5–1.2
					Female: 0.4–0.9
eGFR (mL/min/1.73 m^2^)	35	57	28	52	≥60
Potassium (mEq/L)	5.1 (6.68)*^†^	4.1	5.4	4.1	3.6–5.0
ALT (U/L)	157		183	40	Male: 0–40
					Female: 0–31
AST (U/L)	252	33	408	44	Male: 0–37
					Female: 0–31
ALP (U/L)			729	445	110–350
Total bilirubin (mg/dL)	1.8	0.9	1.4	0.4	0.2–1.2
pH^†^	7.351*		7.406		7.35–7.45
PCO_2_^†^ (mmHg)	29.0*		32.0		35–45
PO_2_^†^ (mmHg)	130.1*		60.7		80–100
HCO_3_^-^^† ^(mmol/L)	15.7*		19.6		24.2–29.8
Base excess^†^(mmol/L)	−8.4*		−4.2		-2.5–2.5
Anion gap^† ^(mmol/L)	16.2*		17.1		10–18
Lactic acid^† ^(mmol/L)	4.62*		4.92		0.4–1.4

Approximately 1 h after admission and 2 h after the above-mentioned blood tests, the patient became apathetic, and communication was difficult. Cold and clammy skin due to poor peripheral perfusion was also observed. At the same time, heart rate decreased to 33 bpm (ECG recording was not obtained), and systemic blood pressure decreased to 70/51 mmHg. Arterial blood gas analysis under oxygen inhalation of 3 L/min via nasal cannula revealed metabolic acidosis, which resulted from lactic acidosis, accompanied by respiratory compensation (Table 1). Plasma potassium level was 6.68 mEq/L. We did not suspect BRASH syndrome yet and started with the continuous intravenous infusion of dopamine hydrochloride (starting at 5 μg/kg/min and increasing to 7 μg/kg/min) for the treatment of hypotension. Moreover, temporary right ventricular pacing at a rate of 70 bpm was performed for the treatment of profound bradycardia. Afterward, the patient’s systemic blood pressure increased. The patient also regained consciousness. In addition, his heart rate increased (Figure 2b) without treatment for hyperkalemia such as intravenous infusion of insulin plus dextrose and oral potassium binding resin. Furosemide was administered intravenously at a dose of 20 mg once daily from Day 2 to Day 4 of hospitalization. Follow-up blood tests showed improvement in liver and kidney function and hyperkalemia (Table 1). The patient was stabilized and weaned off vasopressor agents. The transvenous pacing catheter was removed as well.

**Figure 4:**
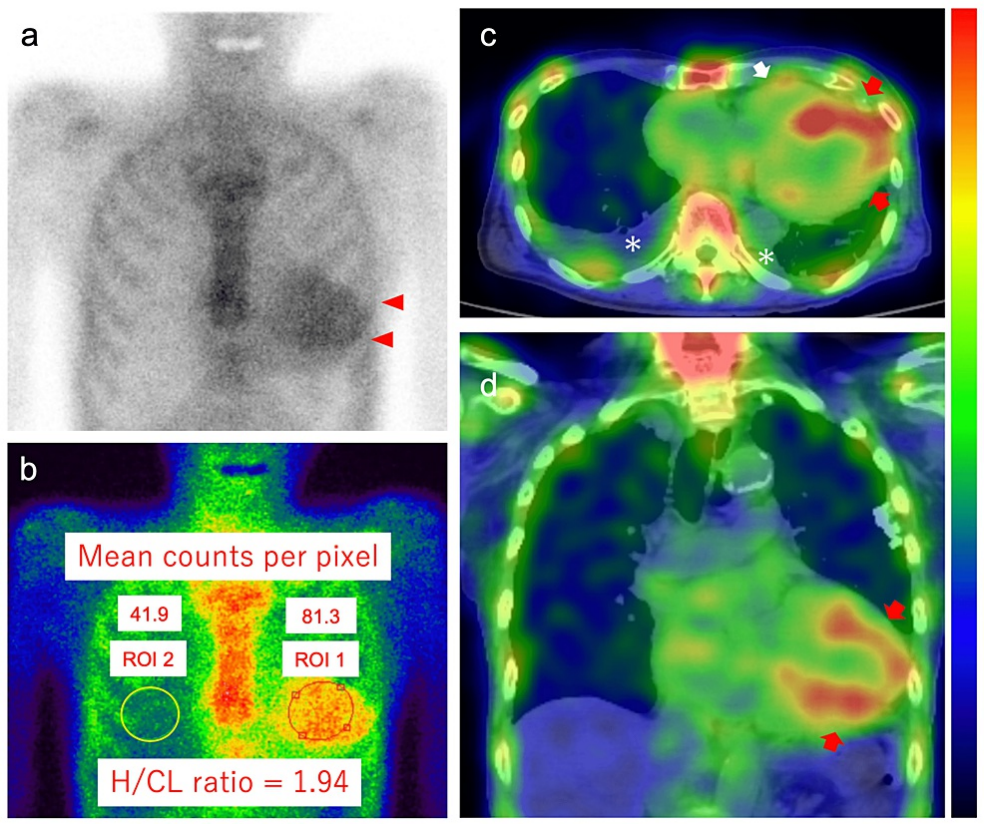
Technetium-99m-pyrophosphate scintigraphy images of Case 1 obtained 2 h after injection of radiotracers Technetium-99m-pyrophosphate planar imaging (anteroposterior view) shows Grade 3 semiquantitative visual scoring of cardiac retention (red arrowheads; high uptake greater than bone): (a) Heart-to-contralateral lung (H/CL) ratio on planar imaging of 1.94, (b) Single-photon emission computed tomographic images with fusion to computed tomographic images show marked tracer uptake in the left (red arrows) and right (white arrow) ventricular myocardium, (c) horizontal plane at the level of the heart, and (d) coronal plane. Regions of interest (ROIs) 1 and 2 indicate the regions of interest positioned to maximize the coverage of the heart without including the adjacent lung and to minimize the overlap with the sternal or focal rib uptake, respectively. Asterisks indicate pleural effusion.

**Figure 5:**
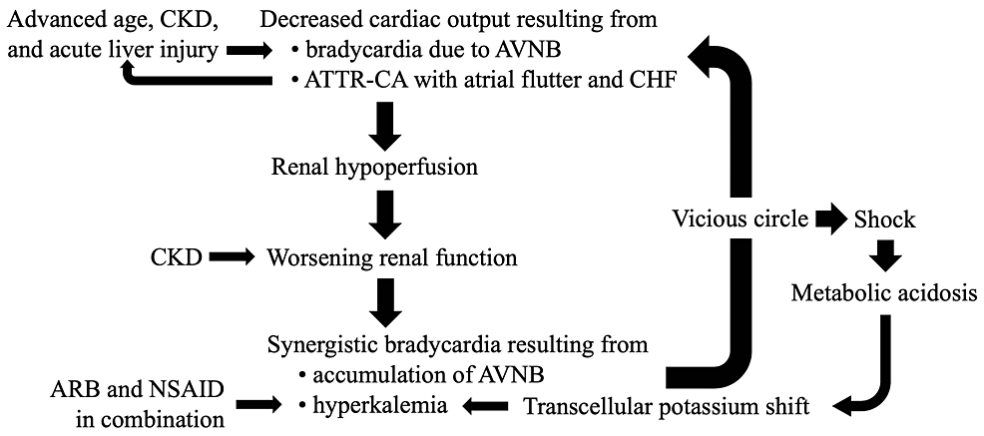
Suspected pathophysiology in Case 1’s bradycardia, renal failure, atrioventricular nodal blockade, shock, and hyperkalemia syndrome ARB, angiotensin II receptor blocker; ATTR-CA, transthyretin cardiac amyloidosis; AVNB, atrioventricular nodal blockade; CHF, congestive heart failure; CKD, chronic kidney disease; NSAID, non-steroidal anti-inflammatory drug.

Technetium-99m-pyrophosphate scintigraphy showed Grade 3 myocardial uptake and a heart/contralateral lung ratio of 1.94. Myocardial uptake was confirmed using single-photon emission computed tomography/computed tomography (SPECT/CT) fusion images (Figure 4). Serum and urine immunofixation tests revealed no immunoglobulin monoclonal proteins, and serum-free light-chain assays showed a normal kappa-lambda ratio of 1.32 (reference range, 0.26-1.65). Therefore, the patient was diagnosed with ATTR cardiac amyloidosis [4]. Transthyretin gene sequencing test was not performed because the patient and his family refused the test and the treatment with tafamidis meglumine, despite the provision of informed consent. The patient was discharged on the 16th hospital day without restarting AV nodal blockers. The suspected pathophysiology in Case 1’s BRASH syndrome is presented in Figure 5.

Case 2

A 90-year-old Japanese woman was brought to Yawatahama City General Hospital by ambulance with the chief complaint of lethargy for several hours. There was no cough or sputum. Diseases being treated at another hospital were long-standing hypertension, dyslipidemia, hyperuricemia, and chronic kidney disease with a serum creatinine level of approximately 0.9 mg/dL and an eGFR of 40 mL/min/1.73 m^2^. Medical history included two episodes of cerebral infarction with little or no residual disability and spinal compression fracture due to osteoporosis. In addition, the patient had shortness of breath on exertion and edema for five or six years and was diagnosed with heart failure with preserved LVEF, but none of the patient’s medical history indicates a high index of suspicion for cardiac amyloidoses such as carpal tunnel syndrome, lumbar spinal stenosis, or biceps tendon rupture. The patient was prescribed carvedilol (10 mg once daily), amlodipine besylate (5 mg once daily), telmisartan (40 mg once daily), nicorandil (7.5 mg three times daily), clopidogrel sulfate (75 mg once daily), febuxostat (20 mg once daily), lansoprazole (10 mg once daily), polaprezinc (75 mg twice daily), and rebamipide (100 mg three times daily) at another hospital. The patient had been taking these medications for months, with no changes in dosage, although home systolic blood pressure had recently decreased to 100 mmHg. Diuretics were not prescribed.

**Figure 6:**
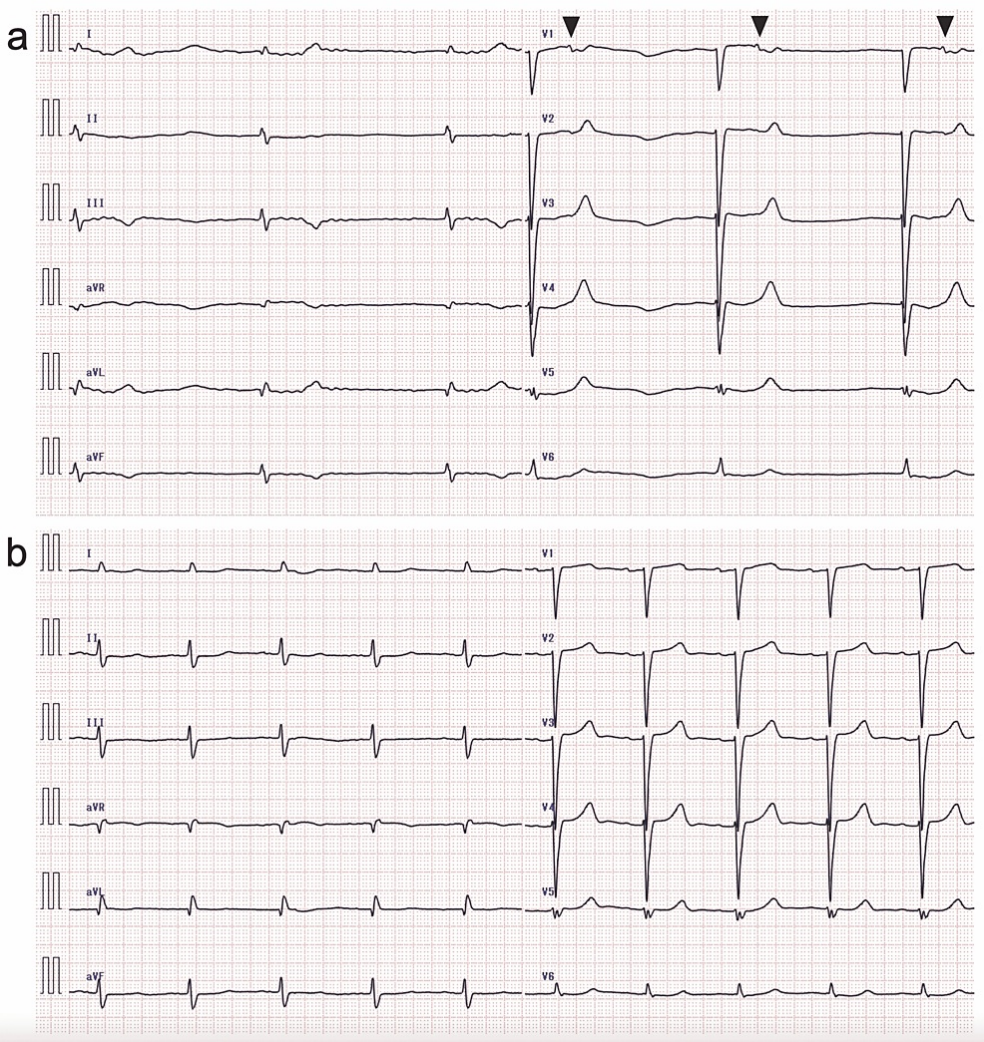
Electrocardiogram (ECG) of Case 2 recorded upon admission (a) and 1 h after intravenous injection of calcium gluconate hydrate (b) The QRS complexes did not change between the two ECG records. Junctional bradycardia with a QRS rate of 29/min was observed upon admission, and sinus rhythm with a QRS rate of 59/min 1 h was observed after intravenous injection of calcium gluconate hydrate. Poor precordial R-wave progression and intraventricular conduction abnormality with a QRS interval of 132 ms are shown. Black arrowheads indicate P waves.

**Figure 7:**
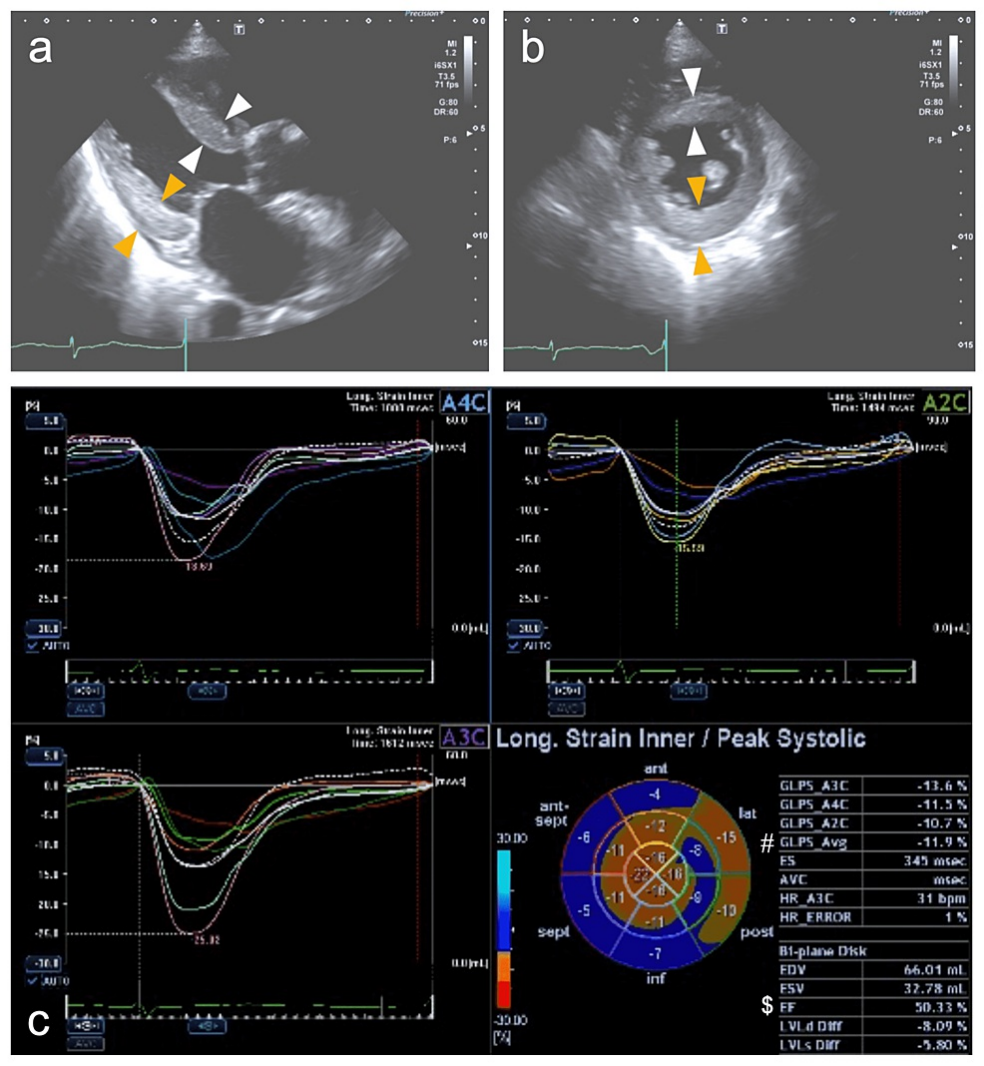
Echocardiograms of Case 2 recorded upon admission A reduced left ventricle with an end-diastolic dimension of 36.5 mm and an increase in the interventricular septum (white arrowheads) and left ventricular (LV) posterior wall (orange arrowheads) of 12.8 and 12.6 mm, respectively, and dilated left atrium with a volume of 65.7 mL/m^2^ are shown (a and b). The bull’s-eye map (with the apex at the center of the color-coding map) illustrates segmental longitudinal LV peak systolic strain values of the 16-segment model generated by speckle-tracking analysis of two-dimensional LV images acquired from apical 2-, 3-, and 4-chamber views (A2C, A3C, and A4C, respectively) and shows a reduced LV ejection fraction (EF) of 50.3% (marked with a dollars sign) and LV global longitudinal peak systolic strain (GLS) value, calculated as the mean of these 16 values, of -11.9% (marked with a hash mark) with an LVEF/|GLS| of 4.2, indicating apical sparing (cutoff, >4.1) (c).

Upon arrival at Yawatahama City General Hospital, the patient had the following vital signs: temperature, 37.8°C; pulse rate, 29 bpm; systemic blood pressure, 76/42 mmHg; oxygen saturation level, 86% on room air. No heart murmurs or rales in the lung fields were audible upon auscultation. The liver was palpable for one and a half finger width along the right midclavicular line below the costal margin, and moderate pretibial edema was observed. Blood tests revealed an elevated high-sensitivity cardiac troponin I value and brain natriuretic peptide value, renal dysfunction, and acute liver injury (Table 1). The patient’s serum potassium level was 5.4 mEq/L. Arterial blood gas analysis on room air revealed hypoxemia and metabolic acidosis, which resulted from lactic acidosis, accompanied by almost appropriate respiratory compensation. Chest radiography revealed mild pulmonary congestion and infiltrating shadows due to bronchopneumonia. ECG revealed junctional bradycardia with a QRS rate of 29/min (Figure 6a). An echocardiogram demonstrated a reduced LV cavity with an end-diastolic dimension of 36.5 mm and an increase in the interventricular septum and LV posterior wall of 12.8 and 12.6 mm, respectively (Figure 7). Hypokinetic wall motions in the entire LV with an LVEF of 50.3% were accompanied by a GLS of -11.9% and an LVEF/|GLS| of 4.2, indicating apical sparing (cutoff value >4.1) [3]. The left atrium, which had a volume of 65.7 mL/m^2^, was dilated. Thus, we suspected the patient had BRASH syndrome, although chronic heart failure caused by cardiac amyloidosis was exacerbated by bronchopneumonia as well, and immediately administered 8.5% calcium gluconate hydrate of 10 mL intravenously, resulting in a dramatic increase in heart rate and systemic blood pressure (52 bpm and 104/58 mmHg, respectively) (Figure 6b). Thereafter, treatment for hyperkalemia, such as intravenous infusion of insulin plus dextrose, in addition to diuretics for heart failure and antibiotics for bronchopneumonia, was started. Carvedilol, amlodipine besylate, and telmisartan were discontinued. Follow-up blood tests showed improvement in liver and kidney function and hyperkalemia (Table 1).

**Figure 8:**
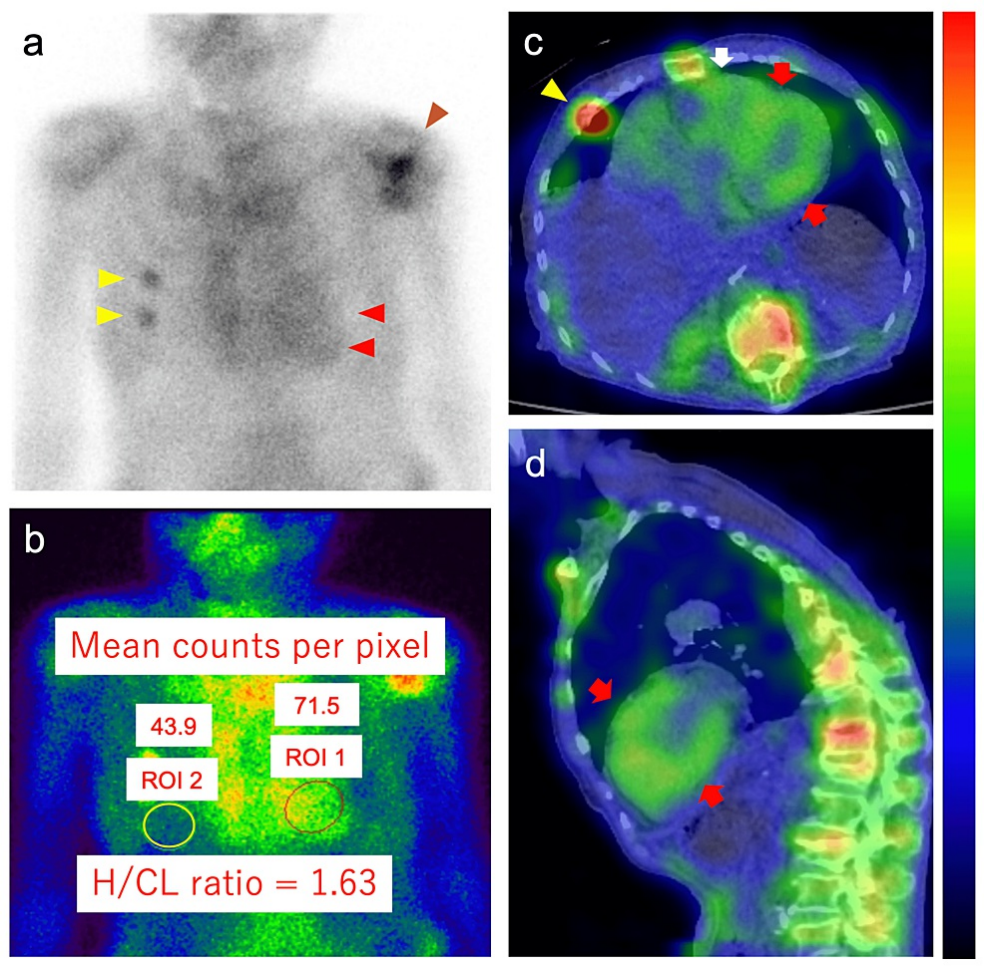
Technetium-99m-pyrophosphate scintigraphy images of Case 2 obtained 2 h after injection of radiotracers Technetium-99m-pyrophosphate planar imaging (anteroposterior view) shows Grade 3 semiquantitative visual scoring of cardiac retention (red arrowheads; high uptake greater than bone) (a) and heart-to-contralateral lung (H/CL) ratio on planar imaging of 1.63 (b). Single-photon emission computed tomographic images with fusion to computed tomographic images show tracer uptake in the left (red arrows) and right (white arrow) ventricular myocardium: (c) horizontal plane at the level of the heart and (d) sagittal plane. Regions of interest (ROIs) 1 and 2 indicate the ROIs positioned to maximize the coverage of the heart without including the adjacent lung and to minimize the overlap with the sternal or focal rib uptake, respectively. Yellow arrowheads and the brown arrowhead indicate the tracer uptake in the right ribs and left shoulder, respectively. Kyphosis is also shown.

Technetium-99m-pyrophosphate scintigraphy showed Grade 3 myocardial uptake, which was confirmed using single-photon emission computed tomography (SPECT-CT) fusion images, and a heart/contralateral lung ratio of 1.63 (Figure 8). Moreover, CT-guided left internal oblique muscle biopsy confirmed ATTR deposition using Congo red staining and immunohistochemical staining, and transthyretin gene sequence testing indicated no variant; thus, the patient was diagnosed with wild-type ATTR cardiac amyloidosis. The patient was discharged on the 15th hospital day without restarting calcium channel-blocking drugs, β-blockers, or angiotensin II receptor blockers.

## Discussion

A constellation of synergistic bradycardia of AV nodal blockades and hyperkalemia secondary to worsening renal function and shock caused by those vicious cycles could be consistent with BRASH syndrome as a cause of rapid deterioration in our two patients. Case 1, in particular, had the following interesting findings: BRASH syndrome developed immediately one day after culprit medications, AV nodal blockers, were started; supposed “pre-BRASH syndrome” preceding BRASH syndrome was observed; blood potassium level increased by approximately 2 mEq/L in just 2 h when “pre-BRASH syndrome” progressed to BRASH syndrome; at the time of BRASH syndrome, temporary right ventricular pacing and dopamine infusion without treatment for hyperkalemia could improve the condition.

In the literature, some patients with BRASH syndrome have serum potassium levels in the first half of 5 mEq/L [1], as shown in our two cases, and the degree of hyperkalemia does not always correlate with the severity of the syndrome. Serum K^+^ is closely regulated physiologically, with normal values ranging from 3.5 to 5.0 mEq/L, and outside of this range, lower and higher values of serum K^+^ have electrophysiological effects that commonly promote cardiac arrhythmias [5]. There is no universally accepted definition of hyperkalemia, and the cutoff of serum potassium concentration as hyperkalemia is usually 5.5 mEq/L [6]. Case 1 began a rather benign hospital course as “pre-BRASH syndrome” without hyperkalemia and shock. Thus, we could not expect the disease to follow the clinical course of a severe illness. However, 2 h after his arrival at Yawatahama City General Hospital, his systemic blood pressure rapidly decreased and his blood potassium level increased by nearly 2 mEq/L, and “pre-BRASH syndrome” progressed toward typical BRASH syndrome at once. As reported by Vishnu et al., the early stages of BRASH syndrome can present without shock [7]. In this setting, systemic blood pressure could be normal because of the pronounced vasoconstrictive response to compensate for bradycardia [1]. In Case 1, mild elevation of blood potassium levels at the time of “pre-BRASH syndrome” might be a warning sign of BRASH syndrome. Moreover, BRASH syndrome might not have occurred if the treatment for bradycardia, including calcium injection, was started during the “pre-BRASH syndrome” period.

It has not yet been clarified when BRASH syndrome develops after culprit medications, AV nodal blockers, are initiated. A report indicated that BRASH syndrome occurred a few days after the AV nodal blocker was started [8], as shown in Case 1, whereas another report indicated that it occurred months or even years later [1], as shown in Case 2. In Case 1, two AV nodal blockers, verapamil and carvedilol, were prescribed. Peak plasma concentrations of verapamil are achieved within 1 h to 2 h of single oral doses in healthy volunteers [9]. The systemic bioavailability of the drug is limited to approximately 20% because of extensive first-pass hepatic extraction and is considerably increased in patients with hepatic injury but is unchanged in patients with renal failure. In addition, verapamil clearance is reduced in patients with hepatic disease and elderly patients. Meanwhile, carvedilol is rapidly absorbed and undergoes extensive first-pass metabolism in the liver [10]. It reaches a peak concentration of 1-2 h after dose and has an elimination half-life of 4-7 h. The drug is metabolized by the liver. The pharmacokinetic profile was not altered in elderly patients or in patients with renal disease. However, the bioavailability of the oral medication is greatly increased in patients with liver disease. In Case 1, BRASH syndrome developed immediately after AV nodal blockade was started because he had multiple precipitating factors to enhance the effects of verapamil and carvedilol, such as advanced age, acute kidney injury on chronic kidney disease, and acute liver injury secondary to congestive heart failure, in the setting of ATTR cardiac amyloidosis associated with atrial flutter. The syndrome might be a distinct entity that lies at the epicenter along a continuum, with isolated hyperkalemia at one end of the spectrum and AV nodal blocker toxicity at the other [11]. In patients with relatively higher contribution to bradycardia by AV nodal blockades than by hyperkalemia, BRASH syndrome develops in a short period after culprit medications are increased in dose or are initially started. In such cases, the treatment includes the management of bradycardia in itself, such as temporary pacing, in addition to the management of hyperkalemia, as shown in case 1 in whom diuretics for the treatment of heart failure expelled potassium from the body. Arif et al. also reported that dopamine infusion, but not calcium injection or insulin plus dextrose injection, improved bradycardia [12]. Temporary right ventricular pacing and dopamine infusion could improve the condition by breaking the vicious cycle (Figure 5). In Case 2, two AV nodal blockers, carvedilol and amlodipine, were prescribed. Amlodipine of approximately 90% is converted to inactive metabolites via hepatic breakdown, and plasma elimination time is prolonged in patients with impaired hepatic function as shown in Case 2, but the pharmacokinetics of amlodipine are not significantly influenced by renal impairment, and patients with renal failure may, therefore, receive the usual initial dose [13]. However, elderly patients show a reduced clearance of amlodipine, with an area under the curve increase of approximately 40%-60%. Amlodipine has an effect on slowing AV conduction, in particular, in combination with β-blockers [14] and is listed as the drug leading to BRASH syndrome [1].

ATTR cardiac amyloidosis has been currently becoming a common disease in elderly individuals with chronic kidney disease and multiple comorbidities requiring polypharmacy and is often associated with heart failure and atrial fibrillation/flutter [15-17]. Patients with ATTR cardiac amyloidosis have a greater propensity for developing disturbed liver function secondary to congestive heart failure [18-19]. In patients with ATTR cardiac amyloidosis, stroke volume is generally decreased and is vulnerable to a decrease in heart rate; thus, the heart rate must be maintained at a high rate [15]. In addition, patients with cardiac amyloidosis may be unusually sensitive to the side effects of common cardiac drugs. Some drugs that are safe for patients with non-cardiac amyloidosis can cause problems in those with cardiac amyloidosis. Non-dihydropyridine calcium channel-blocking drugs, such as verapamil, should be avoided because of negative inotropic effects, particularly in patients with cardiac amyloidosis whose LV function is reduced. The negative chronotropic and dromotropic effects of verapamil may appear strongly in patients with cardiac amyloidosis. Moreover, β-blockers (e.g., carvedilol, metoprolol) probably have little positive effect and may lower the blood pressure excessively. Angiotensin II receptor blockers prescribed to our two patients may also easily induce low cardiac output, leading to hypotension without prognostic benefits in most patients with ATTR cardiac amyloidosis.

## Conclusions

We described two cases of ATTR cardiac amyloidosis diagnosed at the onset of BRASH syndrome. ATTR cardiac amyloidosis has been becoming a common disease in the elderly with chronic kidney disease and multiple comorbidities requiring polypharmacy. Moreover, it is often associated with heart failure, leading to liver dysfunction and atrial fibrillation/flutter. Because patients with ATTR amyloidosis often have multiple intrinsic factors that predispose them to BRASH syndrome, AV nodal blockers must be carefully prescribed to such patients.
